# Is There Really a Loss of Agency in Patients With Apraxia of Tool Use?

**DOI:** 10.3389/fpsyg.2019.00087

**Published:** 2019-02-05

**Authors:** François Osiurak, Mathieu Lesourd, Yves Rossetti, Josselin Baumard

**Affiliations:** ^1^Laboratoire d'Etude des Mécanismes Cognitifs, Université de Lyon, Lyon, France; ^2^Institut Universitaire de France, Paris, France; ^3^Laboratoire de Neurosciences Cognitives, CNRS, LNC, Aix Marseille Univ, Marseille, France; ^4^CNRS, Fédération 3C, Aix Marseille Univ, Marseille, France; ^5^Integrative, Multisensory, Perception, Action, and Cognition Team, Centre de Recherche en Neurosciences de Lyon, INSERM-CNRS-Université de Lyon, Rhône-Alpes, France; ^6^Mouvement, Handicap et Neuro-Immersion, Hospices Civils de Lyon et Centre de Recherche en Neurosciences de Lyon, Rhône-Alpes, France; ^7^UNIROUEN, CRFDP (EA7475), Normandie Univ, Rouen, France

**Keywords:** apraxia, motor action, sense of agency, technical reasoning, tool use

## Introduction

The sense of agency refers to the experience of controlling one's own motor actions and, as a result, external events (Pacherie, 2008; Haggard and Chambon, 2012). Surprisingly, this aspect has been largely overlooked in the literature on apraxia, a disorder affecting skilled and *volitional* movements (De Renzi, 1989). In this context, an outstanding issue is whether loss of agency is an ignored dimension of apraxia—and particularly of apraxia of tool use (see below)—or whether loss of agency and apraxia of tool use are two independent syndromes based on distinct neurocognitive mechanisms. The goal of this Opinion article is to tackle this issue. Note that we will mainly focus here on apraxia of tool use, namely, difficulties in selecting appropriate everyday tools and/or in performing the mechanical action needed to complete a task. This is only one of the manifestations of apraxia, which may also concern the production of symbolic, meaningful, or meaningless gestures (Osiurak and Rossetti, 2017).

## Loss of Agency and Apraxia of Tool Use

The rationale underlying the idea that apraxic patients could experience loss of agency is grounded in classical, neuropsychological models of apraxia. Historically, Liepmann (1908) proposed that tool use is supported by the generation of movement formulae (i.e., the ideation), which are not stored motor programs as commonly thought, but rather images of the action intended (Goldenberg, 2003). There is then a transfer of these movement formulae to the motor innervation. In this framework, patients who fail to generate movement formulae are impaired to use tools. This is the classical description of ideational apraxia: Patients do not know what to do. By contrast, a disconnection between movement formulae and motor innervation leads to ideomotor apraxia: Patients know what to do but not how to do it. A last aspect concerns the deficit of motor innervation (i.e., motor apraxia initially called limb-kinetic apraxia), an aspect we will discuss later in this paper. Although Liepmann's model has been repeatedly updated (Geschwind, 1975; Heilman et al., 1982; Rothi et al., 1991; Cubelli et al., 2000; Buxbaum, 2001, 2017; van Elk et al., 2014), most current, cognitive neuropsychological models are in line with the idea that apraxia of tool use can concern not only the ideational component (i.e., knowing what to do), but also the ability to select the appropriate motor action (i.e., knowing what to do, but not how to do it). Nevertheless, contrary to Liepmann's model, the ideational component is not viewed as the ability to generate a visual image of the action intended, but as the ability to retrieve semantic knowledge about the functional use of everyday tools (hereafter called semantic knowledge). In addition, for Liepmann, movement formulae directly guide the selection of appropriate motor innervatory patterns (i.e., the gesture is reconstructed *de novo* for each use; for a somewhat similar viewpoint, see Osiurak et al., 2011)and ideomotor apraxia is a disconnection syndrome. By contrast, for current models, people store motor programs specifying the kinematic characteristics of movements associated with the use of a specific tool (hereafter called manipulation knowledge). Thus, the issue of (mis-)selection is no longer a disconnection issue but a memory issue. Note that manipulation knowledge can also be considered as an internal model, helpful to control and predict the consequences of one's own motor actions and to compare them to actual outcomes (Sirigu et al., 2003; Buxbaum, 2017).

In this contemporary perspective, three kinds of disorder can be described (i.e., volition, ideation, and selection), even if only two of them (volition and selection) are be labeled as loss of agency (see Pazzaglia and Galli, 2014). The first has been described as a disorder of volition: The patient has difficulties initiating tool-use actions and exhibiting perplexity (e.g., the patient looks hesitatingly at the tools and objects, picks up some of them, puts them down, and so on; De Renzi and Lucchelli, 1988). Given that semantic knowledge (i.e., the ideational component) can be spared, this is a specific disorder of agency: The patient being unable to initiate volitional motor actions (see Pazzaglia and Galli, 2014). The second is a disorder of ideation, that is, of semantic knowledge. This is not an instance of loss of agency because the patient cannot form a mental representation of what to do. The third is a disorder of selection: The patient knows what to do, but is unable to select the appropriate manipulation knowledge (e.g., when asked to pantomime the use of a hammer, the patient performs a first action [rotational wrist movements], then tries to carry out another one [oscillatory elbow movements], and so on). This is also a disorder of agency because the interference, caused by the competition between different degraded motor programs, weakens the subjective perception of controlling one's own motor actions (see Pazzaglia and Galli, 2014).

In broad terms, loss of agency might clearly be an ignored dimension of apraxia of tool use, and contemporary, cognitive models might be particularly suited to develop the study of the underlying mechanisms (Pazzaglia and Galli, 2014). Consistent with this, studies have confirmed the presence of loss of agency in apraxic patients with unilateral hemisphere lesions using self-generated action paradigms (Sirigu et al., 1999, 2004). When reported in these patients, the loss of agency concerns both hands (ipsilesional and contralesional) and occurs independently from elementary sensorimotor disorders (e.g., motor neglect). However, to our opinion, the link between apraxia of tool use and loss of agency is not as obvious as it might appear, and recent evidence has challenged contemporary models of apraxia.

## Volition and Ideation: Two Sides of the Same Coin?

The so-called existence of a selective disorder of volition in apraxia of tool use implies that some patients could have difficulties in initiating tool-use actions without having impaired semantic knowledge (i.e., without a disorder of ideation). This hypothesis presupposes that semantic knowledge supports the ideational component of tool-use actions. However, considerable evidence has indicated that semantic knowledge—commonly assessed with picture-matching tasks—and familiar tool use can be impaired independently (e.g., Negri et al., 2007; Silveri and Ciccarelli, 2009; for a review, see Osiurak and Badets, 2016). In addition, tool-use disorders generally occur in left-brain damaged (LBD) patients presenting lesions within the left inferior parietal lobe (IPL), whereas semantic knowledge involves lesions within the temporal lobes. Taken together, these findings suggest that semantic knowledge cannot be considered as the cognitive basis for the ideation of tool-use actions, as proposed by contemporary, cognitive models of apraxia of tool use, thereby theoretically questioning the aforementioned hypothesis of a disorder of volition without semantic knowledge deficits.

For Liepmann (1908), the ideational component corresponded to the generation of an image of the intended action. Proponents of manipulation-knowledge hypothesis have interpreted it as the visual image of the motor action (i.e., the so-called visuo-kinesthetic engram; e.g., Heilman et al., 1982; Rothi et al., 1991). Nonetheless, another interpretation is that this refers to the visual image of the mechanical action involving tools and objects (e.g., a hammer pounding a nail), an interpretation in line with the technical-reasoning hypothesis (Osiurak et al., 2010; Osiurak and Heinke, 2018; for a somewhat similar viewpoint, see Goldenberg and Hagmann, 1998; Goldenberg and Spatt, 2009). This hypothesis posits that people reason about physical object properties to generate a mental simulation of the mechanical action, appropriate to solve a physical problem. This reasoning is based on mechanical knowledge, that is, knowledge about mechanical and physical principles (e.g., lever, cutting) that might involve the left IPL. A key assumption is that technical reasoning supports the use of both familiar and novel tool use, as confirmed by neuropsychological and neuroimaging studies (for reviews, see Osiurak and Badets, 2016; Reynaud et al., 2016). In this view, patients with impaired technical reasoning can have difficulties not only in initiating tool-use actions (a so-called disorder of volition) but also in performing the appropriate ones (a disorder of ideation). For instance, Osiurak et al. (2013) asked LBD patients with tool-use disorders to solve mechanical problems in using novel tools. Besides severe difficulties in solving the problems, those patients spent a considerable time scrutinizing and grasping tools and objects without initiating tool-use actions, when compared to healthy participants. In other words, those patients were unable to generate a mental simulation of the mechanical solution, preventing them from following any trial-and-error strategy and leading them to perform poorly and to be perplexed. These findings suggest that a so-called disorder of “volition” (i.e., perplexity) may solely be another manifestation of a disorder of ideation (i.e., impaired technical reasoning), but not an instance of loss of agency as suggested earlier. Actually, disorders of “volition” may better suit the clinical description of patients with apathy and apragmatism following damage to the frontal dorsolateral or anterior cingulate cortex, who demonstrate a dissociation between normal motor functions and loss of desire to act (e.g., Devinsky et al., 1995).

## Selection of Motor or Mechanical Actions?

Selective difficulties in selecting appropriate manipulation knowledge (i.e., a disorder of selection) could be viewed as an instance of loss of agency: The patient knowing what to do (no disorder of ideation), but not how to do it (see Pazzaglia and Galli, 2014). This interpretation of patients' difficulties works relatively well if we consider that semantic knowledge supports ideation. However, we have seen earlier that this assumption is questionable. Besides, the technical-reasoning hypothesis offers an alternative idea in suggesting that patients with apraxia of tool use perform incorrect motor actions simply because they are unable to generate an appropriate representation of the correct mechanical action. In a way, the so-called disorder of selection might be not an instance of loss of agency, strictly speaking, but a manifestation of impaired technical reasoning (see below for two other manifestations). Contrary to the technical-reasoning hypothesis, the manipulation-knowledge hypothesis has not been mainly developed from real tool-use tasks, but rather from the pantomime production task (Buxbaum, 2017; Osiurak and Badets, 2017), which consists of asking patients to pretend to use a given tool. In this task, the examiner can only assess patients' motor actions, perhaps increasing the belief that the difficulty is necessarily due to a deficit for selecting the appropriate manipulation knowledge—and, as a result, a potential instance of loss of agency. However, evidence has shown that the demonstration by pantomime is a non-routine, creative task that can involve a plurality of cognitive processes (working memory, semantic memory, communicative skills, technical reasoning; Roy and Hall, 1992; Bartolo et al., 2003; Goldenberg et al., 2003; Baumard et al., 2014; Goldenberg, 2017; Lesourd et al., 2017; Finkel et al., 2018). This multi-determined nature of pantomime is also confirmed by the diversity of brain areas that can be involved in this task (left IPL, left temporal lobe; e.g., Goldenberg and Randerath, 2015; for review, see Niessen et al., 2014), whereas real tool use concerns mainly the left IPL (e.g., Goldenberg and Spatt, 2009). Besides, if demonstration by pantomime is a key task used to observe a deficit of selection and, as a result, a loss of agency, there should be a relatively narrow link between performance in pantomime tasks and sense of agency. A key study in the field did not observe such a link, showing that difficulties in a self-generated action paradigm (supposed to assess the sense of agency) can occur in patients with a perfect score of 100% in a pantomime production task (Sirigu et al., 2004). Taken together, these findings question not only the idea that the so-called disorder of selection is an instance of loss of agency but also, more generally, whether this deficit refers to a clinical reality.

## Apraxia—but not of Tool Use—Can be Accompanied by Loss of Agency

To sum up, apraxia of tool use might not be accompanied by loss of agency, and the so-called manifestations of loss of agency in this syndrome might be nothing else other than the different facets of a disorder of ideation, resulting from impaired technical reasoning. This does not exclude the possibility that apraxia of tool use and loss of agency occur concomitantly because of overlap between the underlying neural substrates (fronto-parietal networks). Nevertheless, the fact that distinct, specific brain areas might be involved in each syndrome confirms the independence of the two syndromes (e.g., left IPL and particularly the supramarginal gyrus for apraxia of tool use vs. right IPL and particularly the angular gyrus for loss of agency; e.g., Farrer et al., 2008). Group studies with brain-damaged patients assessed concomitantly on the two dimensions (i.e., apraxia of tool use and sense of agency) may be particularly useful to test the independence vs. dependence of the two syndromes more directly, not only at a behavioral level but also at a neural level. Perhaps the only apraxic patients experiencing loss of agency might be those suffering from motor apraxia or alien hand sign after lesions within the superior frontal and/or parietal cortex, such as in patients with corticobasal syndrome (Zadikoff and Lang, 2005). These patients frequently and explicitly report that their hand(s) do not do what they want, corroborating a loss of control of their own motor actions (for evidence of loss of agency in this syndrome, see Wolpe et al., 2014). This is perhaps the only divergence we found with Liepmann's conception of apraxia of tool use, because, according to this conception, loss of agency—the patient knows what to do but not how to do it—is supposed to accompany ideomotor apraxia and not motor apraxia.

That being said, another potential cause of loss of agency may lie in body schema disorders. Indeed, patients who have difficulties experiencing their *actions* as their own may also have upstream difficulties experiencing (part of) their *body* as their own, which might be a sufficient and perhaps more parsimonious hypothesis in some cases. This raises issues as to the relationships between body schema and cognition. For example, patients suffering from either partial or global deafferentation following spinal cord injury may demonstrate impairments in laterality judgment tasks, not because of parietal lesions and loss of agency but presumably because of primary loss of body awareness (Ionta et al., 2016). Although the diagnosis of apraxia implies to rule out sensitive and proprioceptive deficits, these latter levels—as well as more complex sensory integration mechanisms—are not systematically tested in studies on the sense of agency. Previous works demonstrated a dissociation between sensory and proprioceptive inputs on the one hand, and perception of self-generated movements on the other hand (e.g., Sirigu et al., 1999), yet body schema is a complex, multi-level process (Sirigu et al., 1991; De Vignemont, 2009). Future research may disentangle the relative contribution of action awareness and body awareness to the performance of apraxic patients in tasks assessing the sense of agency.

## Author Contributions

All authors listed have made a substantial, direct and intellectual contribution to the work, and approved it for publication.

### Conflict of Interest Statement

The authors declare that the research was conducted in the absence of any commercial or financial relationships that could be construed as a potential conflict of interest.
